# 1.2 V Differential Difference Transconductance Amplifier and Its Application in Mixed-Mode Universal Filter

**DOI:** 10.3390/s22093535

**Published:** 2022-05-06

**Authors:** Montree Kumngern, Pichai Suksaibul, Fabian Khateb, Tomasz Kulej

**Affiliations:** 1 Department of Telecommunications Engineering, School of Engineering, King Mongkut’s Institute of Technology Ladkrabang, Bangkok 10520, Thailand; montree.ku@kmitl.ac.th (M.K.); pichai.new@gmail.com (P.S.); 2 Department of Microelectronics, Brno University of Technology, Technická 10, 601 90 Brno, Czech Republic; 3 Faculty of Biomedical Engineering, Czech Technical University in Prague, nám. Sítná 3105, 272 01 Kladno, Czech Republic; 4 Department of Electrical Engineering, Brno University of Defence, Kounicova 65, 662 10 Brno, Czech Republic; 5 Department of Electrical Engineering, Czestochowa University of Technology, 42-201 Czestochowa, Poland; kulej@el.pcz.czest.pl

**Keywords:** mixed-mode filter, universal filter, differential difference transconductance amplifier, analog signal processing

## Abstract

This paper presents a new mixed-mode universal filter based on a differential difference transconductance amplifier (DDTA). Unlike the conventional transconductance amplifier (TA), this DDTA has both advantages of the TA and the differential difference amplifier (DDA). The proposed filter can offer four-mode operations of second-order transfer functions into a single topology, namely, voltage-mode (VM), current-mode (CM), transadmittance-mode (TAM), and transimpedance-mode (TIM) transfer functions. Each operation mode offers five standard filtering responses; therefore, at least twenty filtering transfer functions can be obtained. For the filtering transfer functions, the matching conditions for the input and passive component are absent. The natural frequency and the quality factor can be set orthogonally and electronically controlled. The performance of the proposed topology was evaluated by PSPICE simulator using the 0.18 µm CMOS technology from the Taiwan Semiconductor Manufacturing Company (TSMC). The voltage supply was 1.2 V and the power dissipation of the DDTA was 66 µW. The workability of the filter was confirmed through experimental test by DDTA-based LM13600 discrete-component integrated circuits.

## 1. Introduction

Universal filters are basic electronic blocks that usually provide five filtering responses into a single topology, namely, low-pass (LP), high pass (HP), band pass (BP), band stop (BS), and all pass (AP) filters. The applications such as three crossover network high-fidelity loudspeakers [1,2], touch-tone telephone tone decoders [2], and high-order filters [3] require universal filters as the basic building blocks. Moreover, universal filters can be fabricated as commercial programmable filter-integrated circuits [4]. As a commercially available IC, it is valuable if a single IC can provide a multi-mode filter that depends on the applications of the circuit designer. There are many universal filters available in the open literature, for example, see [5,6,7,8,9,10,11,12,13,14]. Considering input and output signals, these universal filters can be classified as four-mode operations as follows: voltage-mode (VM) filter when both input and output signals are in voltage form [5,6]; current-mode (CM) filter when both input and output signals are in current form [7,8]; transadmittance-mode (TAM) filter when the input signal is in voltage form while the output signal is in current form [9,10,11], and finally transimpedance-mode (TIM) filter when the input signal is in current form while the output signal is in voltage form [12,13,14]. It should be noted that the universal filters in [12,13,14] offer only a single-mode filter.

Recently, universal filters that operate as multi-mode filters into a single topology, the so-called mixed-mode universal filters, have been reported [15,16,17,18,19,20,21,22]. Compared with single-mode universal filters in [5,6,7,8,9,10,11,12,13,14,15], mixed-mode universal filters in [15,16,17,18,19,20,21,22] can provide larger filtering responses. Unfortunately, these mixed-mode universal filters cannot realize four modes of operation into a single topology. There are mixed-mode universal filters that can realize VM, CM, TAM, and TIM filters into a single topology available in the literature [23,24,25,26,27,28,29,30,31,32,33,34,35,36,37,38,39,40,41,42,43,44,45]. However, some of these topologies suffer from some drawbacks as follows:

1. Lack of electronic tunability [24,25,26,27,28,29,34,35,38,39,40,41];
2. Employment of floating passive components [24,25,26,27,28,29,32,35,38,39,41,44,45,46];
3. Active or passive component matching condition [24,25,26,27,28,29,30,31,32,33,34,35,37,39,41,44,46];
4. Input signal matching condition or requirement of a minus-type input signal [30,31,33,34,37,39,45];
5. Input voltage signal being applied via capacitor or resistor [24,25,26,27,28,29,32,34,35,38,39,41,44,45,46]; and
6. Inability to provide at least twenty filtering responses into a single topology [23,24,27,29,33,36,38,40,42,45].

A universal filter that allows electronic tunability can offer some advantages such as the ease of compensation when the natural frequency is deviated by the effect of temperature or process variations, while a universal filter without a floating capacitor and resistor and free from the passive component matching condition is more suitable for integrated circuit implementation. A universal filter that requires a minus-type input signal or an input signal matching condition needs additional circuits such as current-mirror for CM operation or inverting amplifier for VM operation. This requirement defects VM operation because many passive components are usually required, unless the universal filter provides a fully differential structure. Finally, a universal filter that provides at least twenty filtering responses means that each operation mode can realize five standard filtering responses; hence, the full capability of the mixed-mode universal filter can be obtained.

This study focused on a mixed-mode universal filter that could realize VM, CM, TAM, and TIM filters into a single topology. Each operation mode could realize five standard filtering responses; thus, twenty filtering responses could be obtained. The active device, named differential difference transconductance amplifier (DDTA), was used in this study. This device employs high-input impedance terminals with the advantage of input voltage arithmetic operation such as the differential difference amplifier (DDA) [47], and the capability of electronic tuning such as the transconductance amplifier. Thus, a DDTA-based circuit is easy for addition and subtraction of voltage signals and possesses an electronic tuning capability [48,49,50,51]. Unlike the standard differential difference transconductance amplifier that was created by two differential pair DDAs followed by the transconductance amplifier presented in [52], the proposed DDTA is based on one multiple-input differential pair DDA [53,54,55,56] that serves as a differential difference transconductance amplifier followed by a voltage buffer. Therefore, the proposed DDTA could reduce the count of active blocks, power dissipation, and chip area as a result of using the multiple-input MOS transistor (MI−MOST) technique [57]. It is worth noting that the MI-MOST comes with several advantages compared with the multiple-input floating-gate (MIFG) transistor [58]. The MIFG transistor uses the charge conversation principle and hence it is incompatible with modern nanoscale gate-leakage CMOS technologies [59]. The MIFG implementation requires two-polysilicon technology, and the remaining residual charge on its gate causes voltage offset. Therefore, a new DDTA-based mixed-mode universal filter that could provide at least twenty filtering responses of VM, CM, TAM, and TIM filters is presented in this paper. The DDTA uses the MI−MOST technique that offers simplification of its overall structure and a reduction in the power dissipation. The proposed mixed-mode universal filter offers the following advantages such as:

- electronic tuning capability;
- being free from a floating passive component;
- being free from a passive component matching condition;
- lacking a minus-type input signal or an input signal matching condition;
- not applying the input voltage signal via a capacitor or resistor; and
- each operation of VM, TAM, CM and TIM offering five standard filtering responses.

The comparison of the proposed filter with the previous mixed-mode universal filters is shown in Table 1. Compared with [30,31] that have equal active and passive components, the proposed filter is free from active and passive component matching conditions as well as the minus-type input signal requirement. Compared with [43] that offers similar performances, the proposed filter employs fewer components and provides more filtering functions. Compared with [44,45,46] that employ fewer devices, the proposed filter applies the input voltage signal via a high-impedance node whereas the filters in [44,45,46] apply the input voltage signal via a capacitor or resistor.

This paper is organized as follows: in Section 2, the TA-based DDA using MI-MOSTs and the proposed mixed-mode universal filter are presented; Section 3 presents the simulation results and experimental results; and Section 4 concludes the paper.

## 2. Proposed Circuit

### 2.1. Proposed Mixed-Mode Universal Filter

**Figure 1:**
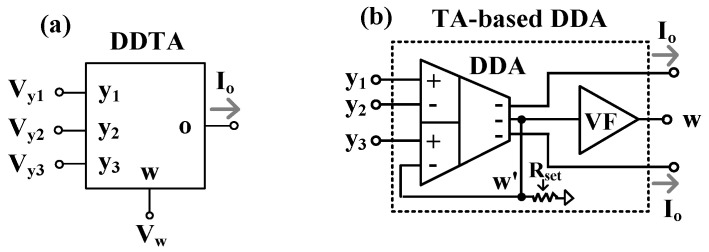
TA-based DDA: (**a**) symbol; (**b**) internal structure.

The symbol of DDTA is shown in Figure 1a. The relationship of the terminals can be expressed by

(1) $$\left.\begin{array}{c} V_{w}=V_{y1}-V_{y2}+V_{y3}\\ I_{o}=G_{m}V_{w} \end{array}\right\}$$

It should be noted that the output $V_{w}$ is the addition and subtraction of inputs $V_{y1}$, $V_{y2}$ and $V_{y3}$, while the output $I_{o}$ is the current that is converted from $V_{w}$ by $G_{m}$, where $G_{m}$ is the internal transconductance of DDTA. Therefore, DDTA included the DDA as an input stage that serves also as a transconductance amplifier (TA) as an output stage. Compared with the differential difference current conveyor transconductance amplifier (DDCCTA) [60], the DDTA structure employs less MOS transistors. Figure 1b shows the internal structure of the proposed DDTA. The voltage follower (VF) circuit was used to avoid the loading effect. Therefore, the w-terminal possessed a low-impedance level that could be directly connected to a low-resistance external load.

**Figure 2:**
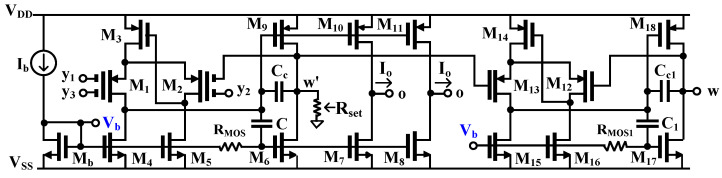
TA-based DDA using MI-MOSTs.

**Figure 3:**
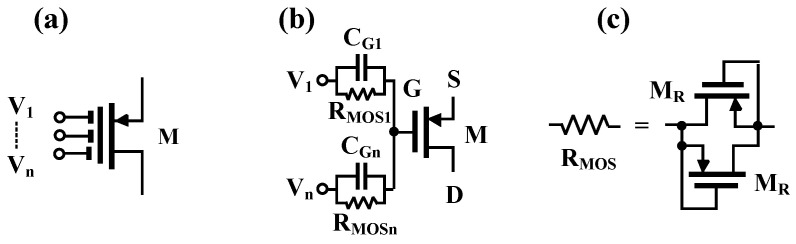
MI-MOST: (**a**) symbol; (**b**) realization; (**c**) realization of the large resistance value.

The structure of DDTA in [52] was developed to the DDTA using MI-MOST as shown in Figure 2. Figure 3a shows the MI-MOST symbol with n number of inputs where the input terminals V_1_, …, V_n_ are coupled to the gate terminal of the conventional MOST by n input capacitors C_G1_, …, C_Gn_. To guarantee the DC operation, the high resistances R_MOS1_, …, R_MOSn_ are connected in parallel to each input capacitor, as shown in Figure 3b. The high resistance $R_{MOS}$ is implemented by two MOSTs (M_R_) operating in the cut-off region as shown in Figure 3c, which offers a minimum area of chip. It is worth noting that the pseudo-resistors shunt the input capacitors for proper DC operation of the input transistor; therefore, there are no floating-gate issues as in the case of the MIFG transistor. However, for AC operation, the input capacitors create a short circuit for the AC signal, the same as in the case of the MIFG technique.

It is worth noting that the multiple input techniques are simply created by a set of parallel capacitors shunted with high-resistance pseudo-resistors (M_R_). This technique can be applied to the gate-, bulk-, gate-bulk (DTMOS), or bulk-quasi-floating-gate terminals of a standard MOS transistor [61].

In Figure 2, the transistors M_1_–M_6_ and M_9_ create the DDA core circuit. The MI-MOST differential pairs M_1_ and M_2_, the transistor M_3_, and the two current sources M_4_ and M_5_ create the differential stage of the DDA. The transistor M_3_ along with M_2_ and M_5_ create a flipped voltage follower (FVF) [62] and it is used to enforce the current of M_3_ (i.e., I_M3_) to be equal to the tail current, same as in the case of the differential stage of the conventional structure. The FVF modifies the gate of M_3_ to ensure equal drain currents for both differential pairs M_1_ and M_2_ [63]. Furthermore, due to the FVF, the minimum voltage supply is the sum of one gate-source and one drain-source voltage ($V_{DD\left(\min\right)}=V_{GS-M3}+V_{DS-M5}$).

Transistors M_6_ and M_9_ form a super class AB second stage [64]. The $R_{\mathrm{MOS}}$ is responsible for the gate DC biasing of the transistor M_6_, whereas the capacitor C delivers the AC signal to this gate. The node $w^{\prime }$ is connected to the input terminal of M_2_, creating negative feedback for obtaining a unity-gain voltage follower. The DDA stability is insured by the compensation capacitor C_c_. The transistors M_12_–M_18_, R_MOS1_, and capacitors C_c1_ and C_1_ are used to work as a voltage follower circuit. The operation is similar to the first stage of DDTA that was previously explained. Therefore, the relationship $V_{w}=V_{y1}-V_{y2}+V_{y3}$ ($V_{w}=V_{w^{\prime }}$) can be obtained. The bias current $I_{b}$ and M_b_ generated the bias voltage $V_{b}$ for M_4_−M_8_ and M_15_−M_17_. The terminal $w^{\prime }$ is connected to a linear adjustable resistor $R_{set}$ that converts the voltage $V_{w^{\prime }}$ to current $I_{w^{\prime }}$. This current is mirrored by M_7_−M_10_ to the o-terminals; thus, $I_{o}=I_{w^{\prime }}$ can be achieved. Additional output current o-terminals can be obtained using complementary transistors such as M_8_ and M_11_. Hence, this part works as a transconductance amplifier. The output current $I_{o}$ is obtained as

(2) $$V_{w^{\prime }}=\left(V_{y1}-V_{y2}+V_{y3}\right)$$

(3) $$I_{o}=\frac{V_{w^{\prime }}}{R_{set}}=\frac{\left(V_{y1}-V_{y2}+V_{y3}\right)}{R_{set}}$$

(4) $$G_{mset}=\frac{1}{R_{set}}=\frac{I_{o}}{\left(V_{y1}-V_{y2}+V_{y3}\right)}$$

Note that the high linearity is achieved due to the linear resistance *R_set_*. The DDA operates in a closed loop, just forming a second-generation current conveyor, with the $w^{\prime }$ output terminal loaded by *R_set_*, and such a configuration can be considered as a transconductance amplifier. However, the attenuation of the input signal by capacitors allows enlarging the input common mode range, as well as the range of linear operation (the range where the so-called hard nonlinearities associated with changing the region of operation of transistors do not appear).

**Figure 4:**
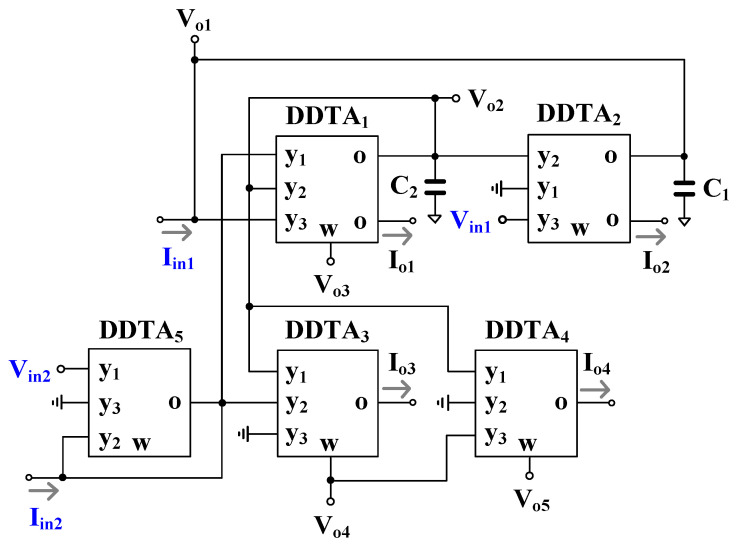
Proposed mixed-mode universal filter using DDTAs.

The proposed mixed-mode universal filter using DDTAs is shown in Figure 4. It consisted of five DDTAs and two grounded capacitors. The variant transfer functions could be obtained by applying the appropriate input signals $V_{in1},\;V_{in2},\;I_{in1}$, and $I_{in2}$ and selecting the appropriate output signals $V_{o1},\;V_{o2},\;V_{o3},\;V_{o4},\;V_{o5},I_{o1}$, and $I_{o2}$. The input voltage which is not used ($V_{in}=0$) should be attached to ground while the input current which is not used ($I_{in}=0$) should be floated. The $G_{msetj}$ ($G_{msetj}=1/R_{setj}$) is the transconductance of ${\mathrm{DDTA}}_{j}$ (j=1, 2, 3, 4, 5). Using (1) and nodal analysis, the output voltages and currents of the proposed mixed-mode universal filter can be expressed by

(5) $$V_{o1}=\frac{\begin{array}{c} G_{mset5}\left(sC_{2}G_{mset2}+G_{mset1}G_{mset2}\right)V_{in1}-G_{mset1}G_{mset2}G_{mset5}V_{in2}\\ -G_{mset5}\left(sC_{2}+G_{mset1}\right)I_{in1}-G_{mset1}G_{mset2}I_{in2} \end{array}}{D\left(s\right)}$$

(6) $$V_{o2}=\frac{\begin{array}{c} G_{mset1}G_{mset2}G_{mset5}V_{in1}+sC_{1}G_{mset1}G_{mset5}V_{in2}\\ -G_{mset1}G_{mset5}I_{in1}+sC_{1}G_{mset1}I_{in2} \end{array}}{D\left(s\right)}$$

(7) $$V_{o3}=\frac{\begin{array}{c} sC_{2}G_{mset2}G_{mset5}V_{in1}+s^{2}C_{1}C_{2}G_{mset5}V_{in2}\\ -sC_{2}G_{mset5}I_{in1}+s^{2}C_{1}C_{2}I_{in2} \end{array}}{D\left(s\right)}$$

(8) $$V_{o4}=\frac{\begin{array}{c} G_{mset1}G_{mset2}G_{mset5}V_{in1}-G_{mset5}(s^{2}C_{1}C_{2}+G_{mset1}G_{mset2})V_{in2}\\ -G_{mset1}G_{mset5}I_{in1}-\left(s^{2}C_{1}C_{2}+G_{mset1}G_{mset2}\right)I_{in2} \end{array}}{D\left(s\right)}$$

(9) $$V_{o5}=\frac{\begin{array}{c} 2G_{mset1}G_{mset2}G_{mset5}V_{in1}-G_{mset5}\left(s^{2}C_{1}C_{2}-sC_{1}G_{mset1}+G_{mset1}G_{mset2}\right)V_{in2}\\ -2G_{mset1}G_{mset5}I_{in1}-\left(s^{2}C_{1}C_{2}-sC_{1}G_{mset1}+G_{mset1}G_{mset2}\right)I_{in2} \end{array}}{D\left(s\right)}$$

(10) $$I_{o1}=\frac{\begin{array}{c} sC_{2}G_{mset1}G_{mset2}G_{mset5}V_{in1}+s^{2}C_{1}C_{2}G_{mset1}G_{mset5}V_{in2}\\ -sC_{2}G_{mset1}G_{mset5}I_{in1}+s^{2}C_{1}C_{2}G_{mset1}I_{in2} \end{array}}{D\left(s\right)}$$

(11) $$I_{o2}=\frac{\begin{array}{c} G_{mset2}G_{mset5}\left(s^{2}C_{1}C_{2}+sC_{1}G_{mset1}\right)V_{in1}-sC_{1}G_{mset1}G_{mset2}G_{mset5}V_{in2}\\ -G_{mset5}\left(s^{2}C_{1}C_{2}+sC_{1}G_{mset1}\right)I_{in1}-sC_{1}G_{mset1}G_{mset2}I_{in2} \end{array}}{D\left(s\right)}$$

(12) $$I_{o3}=\frac{\begin{array}{c} G_{mset1}G_{mset2}G_{mset3}G_{mset5}V_{in1}-G_{mset3}G_{mset5}\left(s^{2}C_{1}C_{2}+G_{mset1}G_{mset2}\right)V_{in2}\\ -G_{mset1}G_{mset3}G_{mset5}I_{in1}-G_{mset3}\left(s^{2}C_{1}C_{2}+G_{mset1}G_{mset2}\right)I_{in2} \end{array}}{D\left(s\right)}$$

(13) $$I_{o4}=\frac{\begin{array}{c} 2G_{mset1}G_{mset2}G_{mset4}G_{mset5}V_{in1}-G_{mset4}G_{mset5}\left(s^{2}C_{1}C_{2}-sC_{1}G_{mset1}+G_{mset1}G_{mset2}\right)V_{in2}\\ -2G_{mset1}G_{mset4}G_{mset5}I_{in1}-G_{mset4}\left(s^{2}C_{1}C_{2}-sC_{1}G_{mset1}+G_{mset1}G_{mset2}\right)I_{in2} \end{array}}{D\left(s\right)}$$

where $D\left(s\right)=s^{2}C_{1}C_{2}G_{mset5}+sC_{1}G_{mset1}G_{mset5}+G_{mset1}G_{mset2}G_{mset5}$. By appropriately applying the input signals ($V_{in1}$, $V_{in2}$, $I_{in1}$, and $I_{in2}$) and choosing the output terminals ($V_{o1}$, $V_{o2}$, $V_{o3}$, $V_{o4}$, $V_{o5}$, $I_{o1}$, $I_{o2}$, $I_{o3}$, and $I_{o4}$), the VM, CM, TAM, and TIM filters can be expressed as in Table 2. It was evident that the proposed filter offers four modes of operation into a single topology. Each mode of operation provides five standard filtering transfer functions; hence, at least twenty transfer functions can be obtained. In addition, several filtering functions can be obtained from the same mode of operation; thus, the proposed topology can provide 36 filtering functions.

It should be noted that some filtering functions offer some advantages such as the gain of transfer function when $V_{in1}$ is the input and $V_{o5}$ is the output for LP of the VM filter, the high-Q filter when $V_{in1}$ = $V_{in2}$ is the input and $V_{o2}$ is the output for BP of the VM filter, and offer both non-inverting and inverting filtering functions for HP of TAM filter.

The natural frequency ($\omega_{o}$) and the quality factor (Q) of the proposed filter can be given as

(14) $$\omega_{o}=\sqrt{\frac{G_{mset1}G_{mset2}}{C_{1}C_{2}}}$$

(15) $$Q=\sqrt{\frac{C_{2}G_{mset2}}{C_{1}G_{mset1}}}$$

From (14) and (15), the parameter $\omega_{o}$ can be adjusted electronically by $G_{mset1}$ and $G_{mset2}$ whereas the parameter Q can be given by $C_{2}/C_{1}$ by keeping $G_{mset1}$ = $G_{mset2}$. Thus, the proposed filter can be electronically controlled for parameter $\omega_{o}$ and orthogonally controlled for parameters $\omega_{o}$ and Q.

It should be noted that the terminals $V_{o3}$, $V_{o4}$, and $V_{o5}$ possess low-output impedance whereas the terminals $I_{o1}$, $I_{o2}$, $I_{o3}$, and $I_{o4}$ possess a high-output impedance, and thus the loads can be connected directly without additional buffer circuit requirements. The terminals $V_{in1}$ and $V_{in2}$ possess a high-input impedance, hence the condition such as $V_{in1}$ = $V_{in2}$ is not required for additional buffer circuits. However, the terminals $V_{o1}$ and $V_{o2}$ do not provide a low-output impedance and the terminals $I_{in1}$ and $I_{in2}$ do not provide a low-input impedance; therefore, the buffer circuits may be required if low-impedance loads are connected and if low-impedance current signals are supplied. In the case of CM and TIM filters, the matching condition is absent and in the case of VM and TAM, the inverting-type input is not used.

### 2.2. Non-Ideality Analysis

Considering non-idealities of DDTA, (1) can be rewritten as

(16) $$\left.\begin{array}{c} V_{w}=\beta_{j1}V_{y1}-\beta_{j2}V_{y2}+\beta_{j3}V_{y3}\\ I_{o}=G_{msetnj}V_{w} \end{array}\right\}$$

where $\beta_{j1}=1-\varepsilon_{j1v}$ and $\varepsilon_{j1v}$ $(\left|\varepsilon_{j1v}\right|\ll 1$) denote the voltage tracking error from $V_{y1}$ to $V_{w}$ of j-th DDTA, $\beta_{j2}=1-\varepsilon_{j2v}$ and $\varepsilon_{j2v}$ $(\left|\varepsilon_{j2v}\right|\ll 1$) denote the voltage tracking error from $V_{y2}$ to $V_{w}$ of j-th DDTA and $\beta_{j3}=1-\varepsilon_{j3v}$ and $\varepsilon_{j3v}$ $(\left|\varepsilon_{j3v}\right|\ll 1$) denote the voltage tracking error from $V_{y2}$ to $V_{w}$ of j-th DDTA.

The non-ideal transconductance gain $G_{msetnj}$ is given by

(17) $$G_{msetnj}\left(s\right)=\left(\frac{\omega_{gmj}}{s+\omega_{gmj}}\right)G_{msetj}$$

where $\omega_{gmj}$ and $G_{msetj}$ denote the first-order pole frequency and the open-loop transconductance gain of j-th DDTA.

The non-ideal transconductance gain of DDTA is caused by the parasitic capacitor and parasitic resistor at o-terminal. In the frequency range that can generate these parasitic parameters, $G_{msetnj}$ can be modified as [65]

(18) $$G_{msetnj}\left(s\right)≅G_{msetj}\left(1-\mu_{j}s\right)$$

where $\mu_{j}=1/\omega_{gmj}$.

The filter in Figure 4 was re-analyzed by using (16), and the denominator of the transfer functions can be rewritten as

(19) $$D\left(s\right)=S^{2}C_{1}C_{2}+SC_{1}G_{msetn1}\beta_{12}+G_{msetn1}G_{msetn2}\beta_{13}\beta_{22}$$

Using (18), (19) becomes

(20) $$\begin{array}{cc} D\left(s\right)= & s^{2}C_{1}C_{2}\left(1-\frac{C_{1}G_{mset1}\beta_{12}\mu_{1}-G_{mset1}G_{mset2}\beta_{13}\beta_{22}\mu_{1}\mu_{2}}{C_{1}C_{2}}\right)\\ & +sC_{1}G_{mset1}\beta_{12}\left(1-\frac{G_{mset1}G_{mset2}\beta_{13}\beta_{22}\mu_{1}+G_{mset1}G_{mset2}\beta_{13}\beta_{22}\mu_{2}}{C_{1}G_{mset1}\beta_{12}}\right)+G_{msetn1}G_{msetn2}\beta_{13}\beta_{22} \end{array}$$

From (20), the non-idealities of the DDTAs affect the circuit characteristics which depart from ideal values. The parasitic effects from the DDTA could be made negligible by satisfying the following condition:

(21) $$\frac{\beta_{12}C_{1}G_{mset1}\mu_{1}+\beta_{13}\beta_{22}G_{mset1}G_{mset2}\mu_{1}\mu_{2}}{C_{1}C_{2}}\ll 1$$

(22) $$\frac{\beta_{13}\beta_{22}G_{mset1}G_{mset2}\mu_{1}-\beta_{13}\beta_{22}G_{mset1}G_{mset2}\mu_{2}}{\beta_{12}C_{1}G_{mset1}}\ll 1$$

Therefore, the non-ideal natural frequency ($\omega_{on}$) and the non-ideal quality factor ($Q_{n}$) can be expressed, respectively, by

(23) $$\omega_{on}=\sqrt{\frac{G_{mset1}G_{mset2}\beta_{13}\beta_{22}}{C_{1}C_{2}}}$$

(24) $$Q_{n}=\frac{1}{\beta_{12}}\sqrt{\frac{C_{2}G_{mset2}\beta_{13}\beta_{22}}{C_{1}G_{msetn1}}}$$

The sensitivity of the $\omega_{on}$ and $Q_{n}$ with respect to circuit components and non-ideal parameters can be expressed as follows:

(25) $$S_{G_{mset1}}^{\omega_{on}}=S_{G_{mset2}}^{\omega_{on}}=S_{\beta_{13}}^{\omega_{on}}=S_{\beta_{22}}^{\omega_{on}}=-S_{C_{1}}^{\omega_{on}}=-S_{C_{2}}^{\omega_{on}}=\frac{1}{2}$$

(26) $$S_{\beta_{12}}^{Q_{n}}=-1$$

(27) $$S_{C_{2}}^{Q_{n}}=S_{G_{mset2}}^{Q_{n}}=S_{\beta_{13}}^{Q_{n}}=S_{\beta_{22}}^{Q_{n}}=-S_{C_{1}}^{Q_{n}}=-S_{G_{mset1}}^{Q_{n}}=-\frac{1}{2}$$

It can be expressed from (25)–(27) that the proposed filter showed good active and passive sensitivities because all the sensitivities were within unity in magnitude.

## 3. Results

### 3.1. Simulation Results

The DDTA in Figure 2 was designed using a 1.2 V voltage supply (V_DD_ = −V_SS_ = 0.6 V) and 5 µA bias current. The circuit consumed 66 µW of power. The PSPICE simulation was used to simulate the circuit using a 0.18 µm CMOS technology from TSMC. The parameters of the components and the simulated performances of the used DDTA are shown in Table 2 and Table 3, respectively.

**Table 2:** Parameters of the components of DDTA in Figure 2.

Transistor	W/L (µm/µm)
M_1_, M_2_, M_13_, M_12_	9 × 9/0.3
M_3,_ M_14_	15/0.3
M_b_, M_4_, M_5_, M_15_, M_16_	12/3
M_6_, M_7_, M_8_, M_17_	2 × 12/3
M_9_, M_10_, M_11_, M_18_	2 × 25/2
M_R_	4/5
C_G_ = 0.5 pF, C_c_ = C = 2.6 pF	

**Figure 5:**
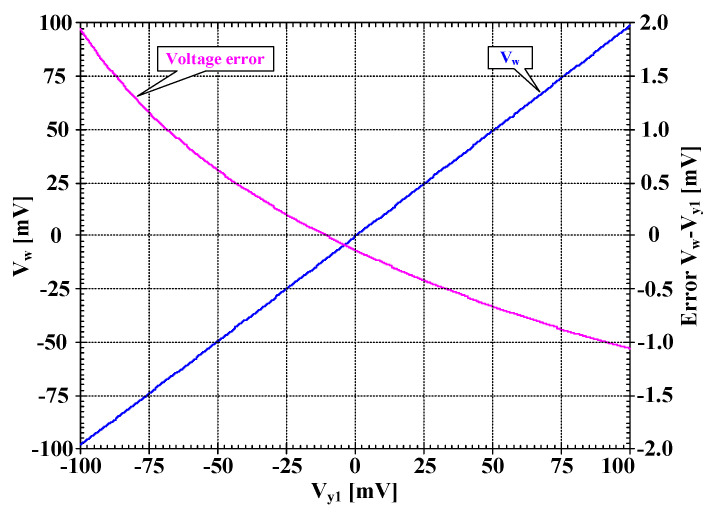
The simulated large signal DC transfer characteristic V_W_ = f(V_y1_) and the corresponding error.

**Figure 6:**
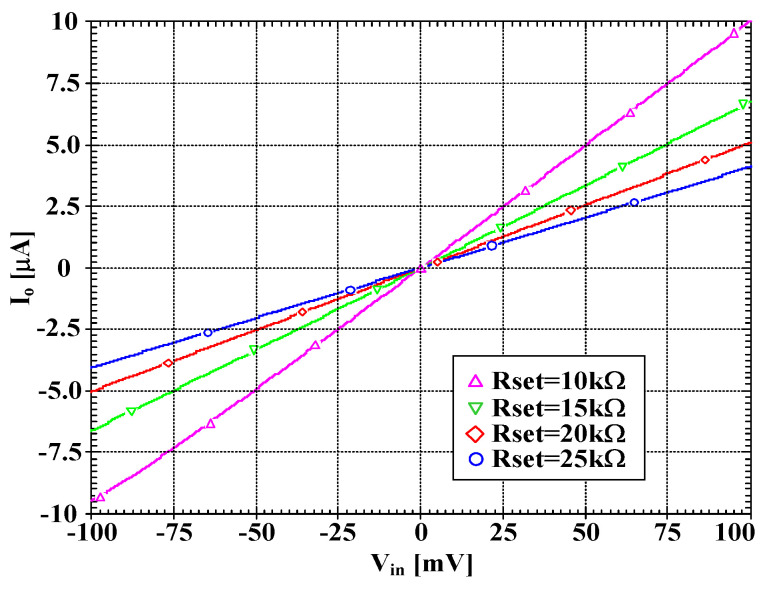
The simulated large-signal DC transfer characteristic I_o_ = f(V_in_) for different values of R_set_.

Figure 5 shows the relation between voltages $V_{w}$ and $V_{y1}$ with $R_{set}$ = 15 kΩ and its voltage error. At $V_{y1}$ = 0 mV, the voltage error was −0.13 mV and at $V_{y1}$ = ±100 mV, the voltage error was less than 2 mV. To show the voltage-to-current converter of DDTA, the voltage $V_{in}$ ($V_{in}$ = $V_{in+}-V_{in-}$) was applied to the input, and the current at o-terminal was measured. Figure 6 shows the relation between I_o_ and V_in_ with different values of $R_{set}$ ($R_{set}$ = 10, 15, 20, 25 kΩ). The transconductances $G_{mset}$ of DDTA can be given by $1/R_{set}$ ($G_{mset1}$ = $1/R_{set}$). The simulated performances of DDTA in Figure 2 are summarized in Table 3.

The proposed mixed-mode filter in Figure 4 was designed for obtaining 1 kHz of the natural frequency. The capacitors $C_{1}=C_{2}=$10 nF and $R_{set1}=R_{set2}=R_{set3}=R_{set4}=R_{set5}=$ 15 kΩ. These $R_{set}$ resistors can be integrated on chip using a high-resistance poly resistor; however, the high value 10 nF capacitors should be off-chip.

**Figure 7:**
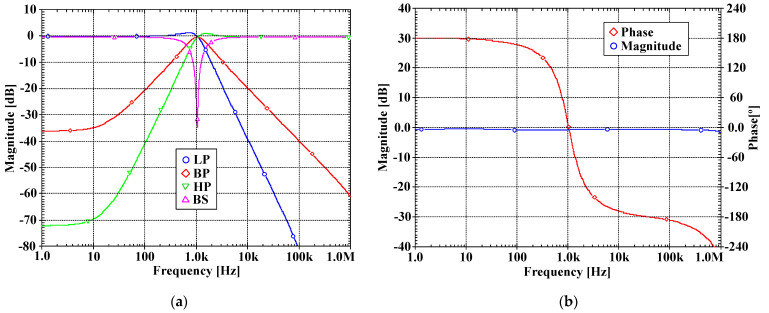
The simulated frequency responses of the VM filter: (**a**) LP, BP, HP, BS filters; (**b**) AP filter.

**Figure 8:**
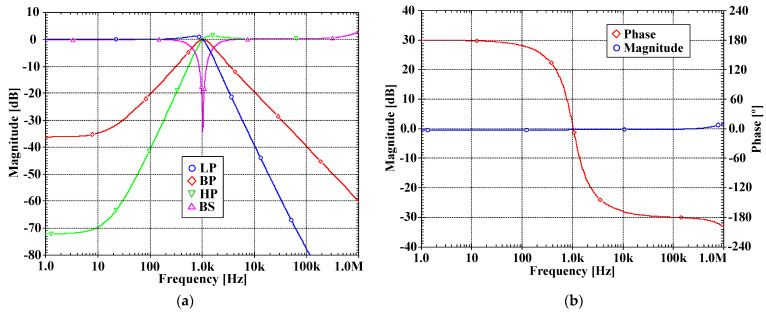
The simulated frequency responses of the CM filter: (**a**) LP, BP, HP, BS filters; (**b**) AP filter.

**Figure 9:**
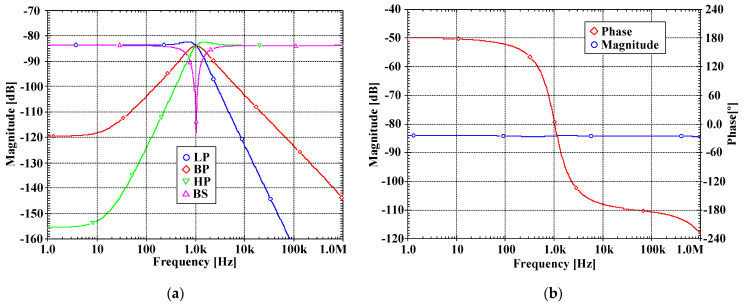
The simulated frequency responses of the TAM filter: (**a**) LP, BP, HP, BS filters; (**b**) AP filter.

**Figure 10:**
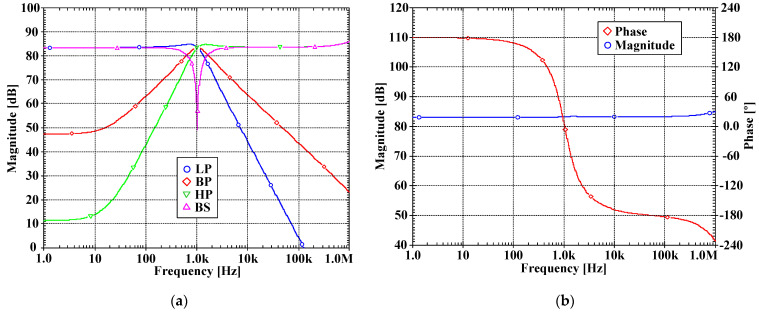
The simulated frequency responses of the TIM filter: (**a**) LP, BP, HP, BS filters; (**b**) AP filter.

Figure 7a, Figure 8a, Figure 9a and Figure 10a show, respectively, the simulated magnitude frequency responses of the LP, HP, BP, and BS responses of the VM, CM, TAM, and TIM filters. The natural frequency of these results was 1.04 kHz. The simulated magnitude and phase characteristics of the AP filter of the VM, CM, TAM, and TIM filters are shown respectively in Figure 7b, Figure 8b, Figure 9b and Figure 10b. The total power consumption of the filter was 330 µW. It can be confirmed from Figure 7, Figure 8, Figure 9 and Figure 10 that the proposed mixed-mode filter provides five standard filtering responses of VM, CM, TAM, and TIM filters.

**Figure 11:**
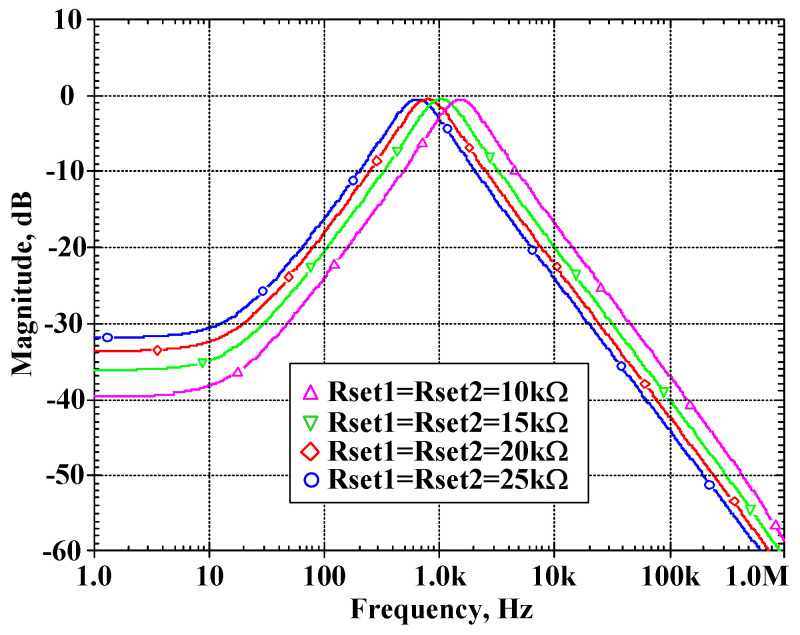
The simulated frequency responses of the VM BP filter when variation in f_o_ by $R_{set}$ ($R_{set1}$ = $R_{set2}$ = 10 kΩ, 15 kΩ, 20 kΩ and 25 kΩ while $R_{set3}$ = $R_{set4}$ = $R_{set5}$ = 15 kΩ).

To confirm that the proposed filter provided electronic tuning ability, the BP filter was simulated by adjusting $R_{set1}$ = $R_{set2}$ = 10 kΩ, 15 kΩ, 20 kΩ, 25 kΩ while $R_{set3}$ = $R_{set4}$ = $R_{set5}$ = 15 kΩ. Figure 11 shows the center frequency of 0.64 kHz, 0.79 kHz, 1.04 kHz, and 1.51 kHz when the resistance $R_{set1}$ = $R_{set2}$ was 25 kΩ, 20 kΩ, 15 kΩ, and 10 kΩ, respectively.

**Figure 12:**
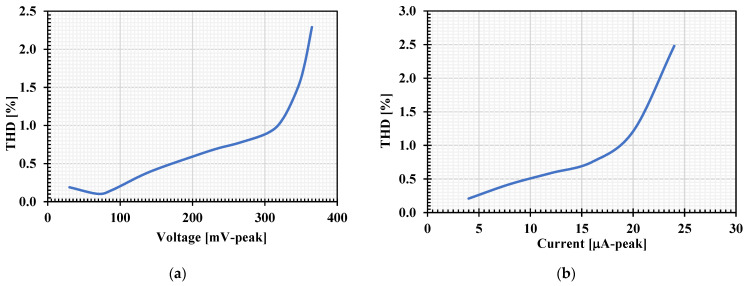
The simulated THD of the LP filters with different amplitude of input signal at 100 Hz: (**a**) VM filter; (**b**) CM filter.

The total harmonic distortion (THD) of the LP response of VM and CM filters was investigated by applying the single-tone input signal of 100 Hz to the input. The simulated THDs of VM and CM filters with different amplitudes are respectively shown in Figure 12a,b. The THD was less than 1.09% for input amplitude of 325 mV (peak) of the VM filter and the THD was less than 1.21 for input amplitude of 20 μA (peak) of the CM filter. The RMS output noise of the LP filter integrated in the bandwidth of 1 kHz was performed and the value of this noise was 150 µV. Thus, the dynamic range for 1.09% THD was 63.69 dB.

**Figure 13:**
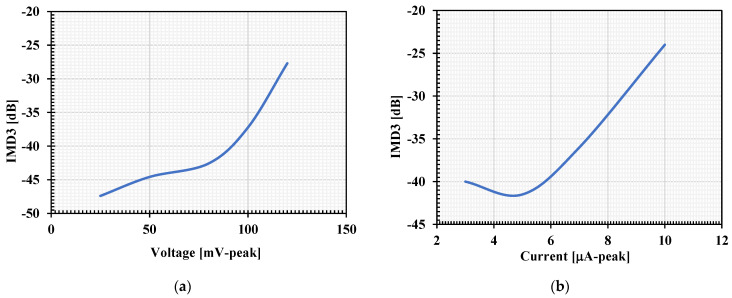
The simulated IMD3 versus the input signal for the BP filters: (**a**) voltage (V_in_-peak); (**b**) voltage (I_in_-peak).

The proposed filter was investigated by applying two tones closely spaced in frequency into the input of the BP filter and the third-order distortion products (IMD3s) produced by the circuit nonlinearity were determined. In this case, the IMD3 of the VM and CM filters was investigated by applying the first tone with a sine wave frequency of 0.9 kHz and the second tone with 1.1 kHz. The simulated IMD3s of the VM and CM filters are respectively shown in Figure 13a,b. The IMD3 was around −37.23 dB for 100 mV (peak) of the VM filter and the IMD3 was around −36 dB for 7 μA (peak) of the CM filter.

**Figure 14:**
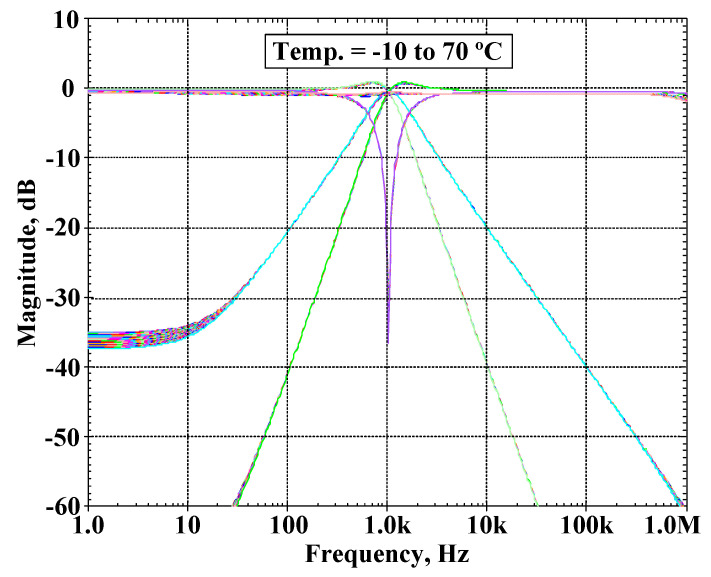
The simulated magnitude frequency responses of the universal filter with temperature variation.

**Figure 15:**
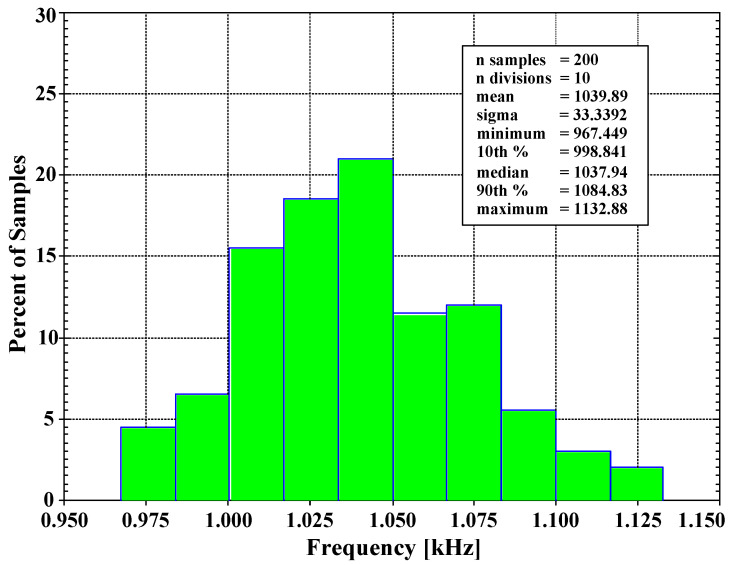
The histogram of the cutoff frequency of the universal filter with 200 runs of MC analysis.

The VM filter was used to test its temperature performance. The simulated magnitude frequency responses of the LP, BP, HP, BS, and AP filter when the temperature was varied from −10 to 70 °C are shown in Figure 14. The proposed filter was also investigated using a Monte Carlo analysis by assuming that the fluctuation of the natural frequency changes caused by deviation of the capacitors and the threshold voltage of the MOS transistor. The BP response of the VM filter was simulated by setting 5% tolerances of the capacitors C_1_ and C_2_ and 5% variations of the transistor threshold voltage at 1.04 kHz, Q ≅ 1, and 200 Gaussian distribution runs. Figure 15 shows the derived histogram of the natural frequency which expressed that the standard deviation (σ) of f_o_ was 33.339 Hz and the maximal and minimal values of f_o_ were 1.132 kHz and 0.967 kHz, respectively.

### 3.2. Experimental Results

**Figure 16:**
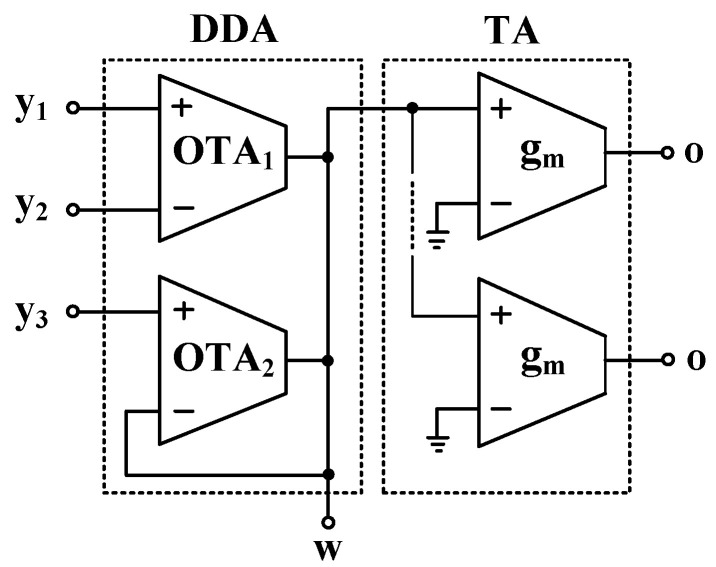
OTA-based DDTA [52].

The proposed mixed-mode universal filter was also tested experimentally to confirm its functionality. The simulation results based on the macro model and the measured results are included for comparison. The DDTA was realized using OTAs as shown in Figure 16 [52]. The prototype circuit was realized using commercially available integrated circuit LM13700N that consists of two current-controlled transconductance amplifiers. Note the benefit of the MI-MOST on the TA-based DDA in Figure 2 in simplifying the CMOS structure and reducing the number of ICs needed to build the filter application. For instance, to create the multiple input (y_1_, y_2_, and y_3_) of the DDA in Figure 16, two transconductance amplifiers (OTA_1_, OTA_2_) are needed and another two OTAs are needed to construct the TA, hence two LM13700Ns are needed for each DDTA.

**Figure 17:**
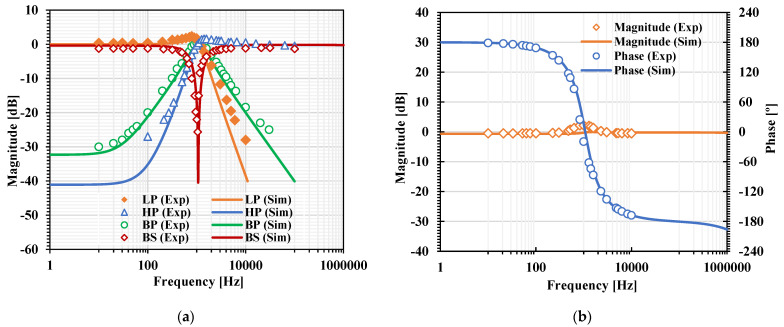
Experimental frequency responses of the VM filter: (**a**) LP, BP, HP, BS filters; (**b**) AP filter.

**Figure 18:**
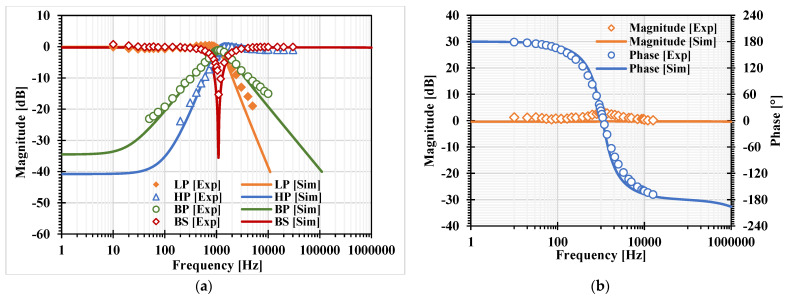
Experimental frequency responses of the CM filter: (**a**) LP, BP, HP, BS filters; (**b**) AP filter.

**Figure 19:**
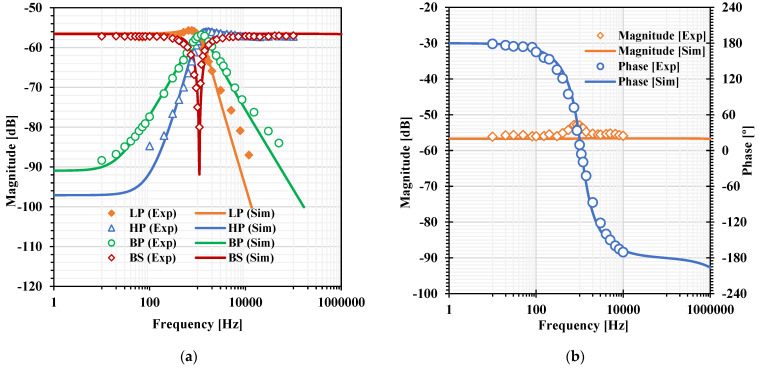
Experimental frequency responses of the TAM filter: (**a**) LP, BP, HP, BS filters; (**b**) AP filter.

**Figure 20:**
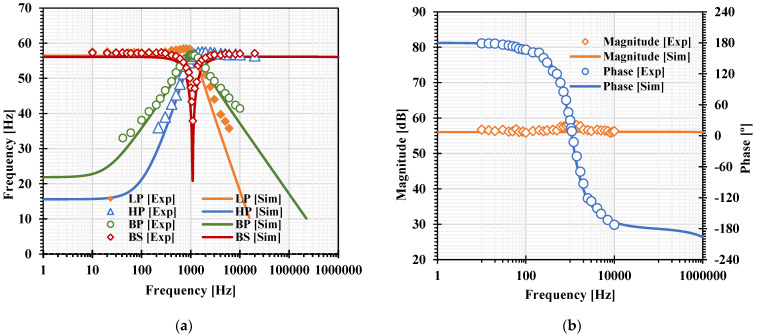
Experimental frequency responses of the TIM filter: (**a**) LP, BP, HP, BS filters; (**b**) AP filter.

For measurement setup, the supply voltage was ±5 V and the capacitances C_1_ and C_2_ were 220 nF. The Agilent Technology DSOX 1102G oscilloscope was used for supplying the sinusoidal input signal and measuring the output waveforms. The transconductances $g_{m1}$ = $g_{m2}$ = $g_{m3}$ = $g_{m4}$ = $g_{m5}$ = 1.51 mS were designed to obtain the mixed-mode filter with the natural frequency of 1.09 kHz and the quality factor of 1 (Q ≅ 1). Figure 17a, Figure 18a, Figure 19a and Figure 20a show the experimental frequency responses of the LP, HP, BP, and BS responses of the VM, CM, TAM, and TIM filters, respectively. Figure 17b, Figure 18b, Figure 19b and Figure 20b show the experimental frequency response of magnitude and phase characteristics of the AP responses of the VM, CM, TAM, and TIM filters, respectively. To measure the frequency responses of TAM filter, a resistor was used to convert the output current to voltage, and the voltage according to this resistance was calculated to the output current for plotting. In case of CM and TIM filters, the high resistances (i.e., $R_{in}$ ≫ 662 Ω) were used to convert the input voltage to the input current at input terminals and convert the output current to the output voltage output terminals. The voltage according to the resistances was calculated as currents for plotting.

**Figure 21:**
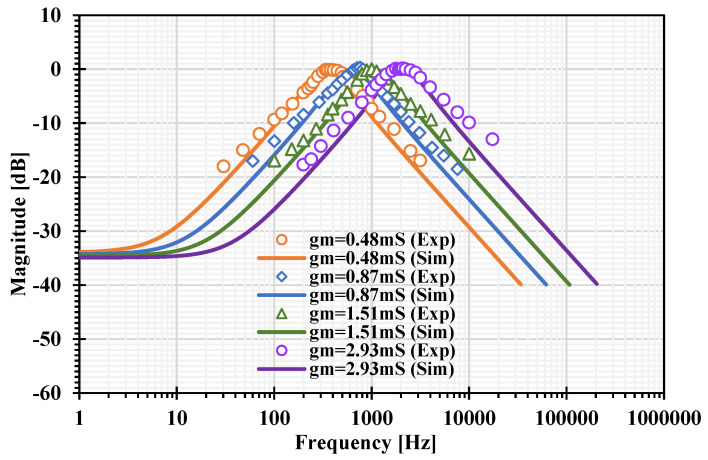
The experimental frequency responses of the BP response of the VM filter with different transconductances.

The experimental frequency responses of the BP response of the VM filter with different transconductances ($g_{m}$ = 0.48 mS, 0.87 mS, 1.51 mS, and 2.93 mS) are shown in Figure 21. This result was used to confirm that the proposed mixed-mode filter provides an electronic tuning ability without drubbing the quality factor. The Experimental setup of the universal filter is shown in Figure S1 in the Supplementary Materials.

## 4. Conclusions

A new mixed-mode universal filter using five DDTAs and two grounded capacitors was shown in this paper. The proposed filter offers 36 filtering responses into a single topology using the DDTA-based circuit. The natural frequency and the quality factor can be set orthogonally and electronically controlled. The performance of the proposed filter was evaluated in PSPICE simulation using the TSMC 0.18 µm CMOS technology and investigated by experiment tests using LM13600 discrete component integrated circuit as DDTAs. The simulation results were in agreement with the experimental results.

## Figures and Tables

**Table 1:** Comparison the proposed filter with the previous mixed-mode universal filter.

Ref.	No. of Device	Power Supply	No. of C & R	Obtaining Function	PD [mW]	THD of LP [%]	BW [kHz]	(i)	(ii)	(iii)	(iv)	(v)	(vi)
[23] 2003	4-CCCII	-	2 & 0	14	-	-	-	Yes	Yes	Yes	Yes	Yes	No
[24] 2004	5-CCII	-	2 & 7	12	-	-	-	No	No	No	Yes	No	No
[25] 2005	4-CFOA	±12 V	2 & 9	20	-	-	112.5	No	No	No	Yes	No	Yes
[26] 2006	3-CCII	±12 V	3 & 4	20	-	-	-	No	No	No	Yes	No	Yes
[27] 2006	3-FTFN	-	2 & 3	11	-	-	31.8	No	No	Yes	Yes	No	No
[28] 2007	2-DDCC	±1.25 V	2 & 4	20	-	-	4.973 × 10^3^	No	No	No	Yes	No	Yes
[29] 2008	1-FDCCII	±1.25 V	2 & 3	17	-	-	3.316 × 10^3^	No	No	No	Yes	No	No
[30] 2009	5-OTA	±1.65 V	2 & 0	24	30.95	-	1 × 10^3^	Yes	Yes	No	No	Yes	Yes
[31] 2010	5-OTA	±1.25 V	2 & 0	20	-	0.777@400 mV_pp_	1.591 × 10^3^	Yes	Yes	No	No	Yes	Yes
[32] 2010	2-CCCII	±2.5 V	2 & 1	20	-	<5@500 μA_pp_	1.27 × 10^3^	Yes	No	No	Yes	No	Yes
[33] 2011	3-CCCCTA	±1 V	2 & 0	16	4.84	-	1.06 × 10^3^	Yes	Yes	No	No	Yes	No
[34] 2011	3-DDCC	±1.25 V	2 & 3	30	-	0.723@60 μA_pp_	3.978 × 10^3^	No	Yes	No	No	Yes	Yes
[35] 2011	3-DDCC	±1.25 V	2 & 4	20	-	-	3.978 × 10^3^	No	No	No	Yes	No	Yes
[36] 2012	4-MOCCCII	±2.5 V	2 & 0	12	-	-	-	Yes	Yes	Yes	Yes	Yes	No
[37] 2013	4-MOCCCII	±1.25 V	2 & 0	20	-	0.5@300 μA_pp_	-	Yes	Yes	No	No	Yes	Yes
[38] 2015	2-CCII	±1.25 V	2 & 2	11	-	-	2 × 10^3^	No	No	Yes	Yes	No	No
[39] 2016	1-FDCCII, 1-DDCC	±0.9 V	2 & 6	46	-	2.2@300 mV_pp_	1.591 × 10^3^	No	No	No	No	No	Yes
[40] 2016	2-DVCC	±1.25 V	2 & 3	14	-	-	3.978 × 10^3^	No	Yes	Yes	Yes	Yes	No
[41] 2016	2-FDCCII	±0.9 V	2 & 5	25	-	0.971@200 mV_pp_	1.591 × 10^3^	No	No	No	Yes	No	Yes
[42] 2017	3-CCCCTA	±0.9 V	2 & 0	18	1.99	2.16@500 mV_pp_	3.183 × 10^3^	Yes	Yes	Yes	Yes	Yes	No
[43] 2017	6-MI-OTA	±0.5 V	2 & 0	20	0.075	2@50 mV_pp_	1.5 × 10^3^	Yes	Yes	Yes	Yes	Yes	Yes
[44] 2020	2-EXCCTA	±1.25 V	2 & 4	20	-	<5@520 mV_pp_	7.622 × 10^3^	Yes	No	No	Yes	No	Yes
[45] 2021	1-EX-CCCII	±0.5 V	2 & 1	17	1.35	0.2@520 mV_pp_	23 × 10^3^	Yes	No	Yes	No	No	No
[46] 2021	1-VD-EXCCII	±1.25 V.	2 & 3	20	5.76	<7.5@650 mV_pp_	8.084 × 10^3^	Yes	No	No	Yes	No	Yes
This study	5-DDTA	1.2 V	2 & 0	36	0.33	1.09@650 mV_pp_	1.04	Yes	Yes	Yes	Yes	Yes	Yes

Note: PD = power dissipation, THD = total harmonic distortion, and BW = bandwidth.

**Table 3:** Simulated parameters of used DDTA.

Parameters	Simulated Value
Technology	0.18 μm
Supply voltage	1.2 V (±0.6 V)
Static power consumption	66 μW
Transconductance	1/R_set_
−3 dB bandwidth	
V_w_/V_y1_, V_w_/V_y2_, V_w_/V_y3_	2.4 MHz
I_o_/V_y1_ (R_set_ = 15 kΩ)	6.4 MHz
Voltage gain: V_w_/V_y1_, V_w_/V_y2_, V_w_/V_y3_	0.988
DC voltage range (R_set_ = 15 kΩ)	±100 mV
DC offset	−0.13 mV
R_w_&L_w_	1.25 kΩ & 0.4 mH
R_o_//C_o_	947.78 kΩ//0.22 pF

## Supplementary Materials

The following supporting information can be downloaded at: https://www.mdpi.com/article/10.3390/s22093535/s1, Figure S1: Experimental setup of the universal filter.
