# Supplementary material for: 1.2 V Differential Difference Transconductance Amplifier and Its Application in Mixed-Mode Universal Filter
Source: Sensors (Basel). 2022 May 6;22(9):3535. doi: 10.3390/s22093535 (PMC9101344; doi:10.3390/s22093535)
Supplement: Supplementary file 1 [file sensors-22-03535-s001.zip › sensors-1632365-supplementary.pdf]

Supplementary Materials

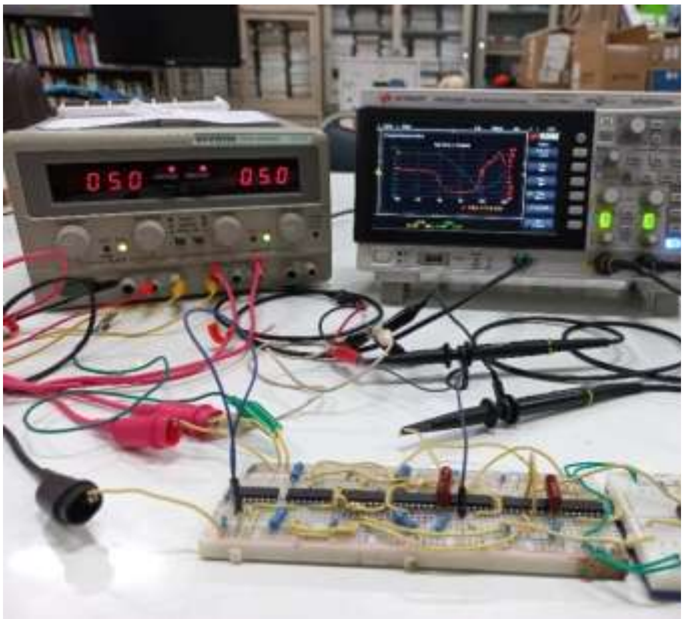

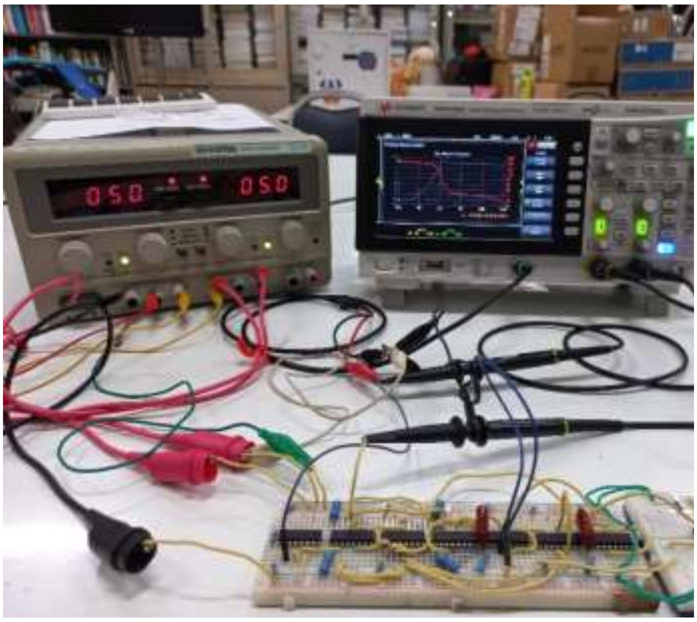

LP response of VM filter

HP response of VM filter

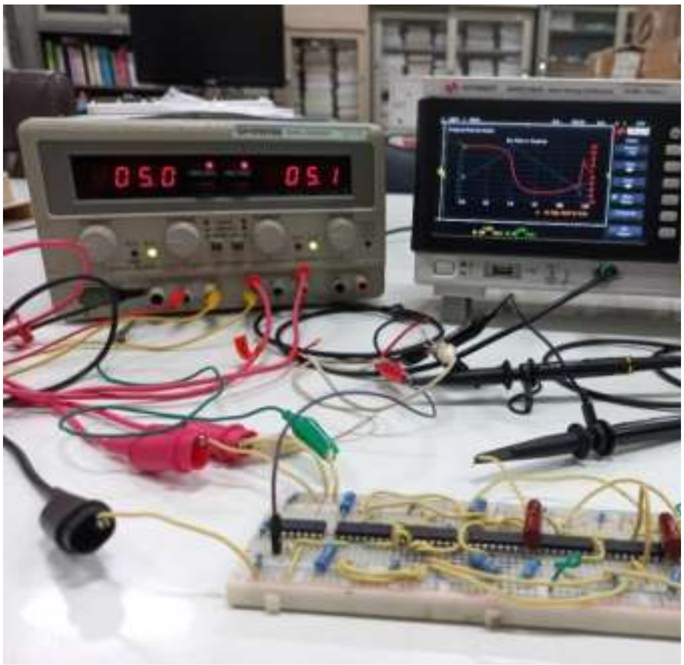

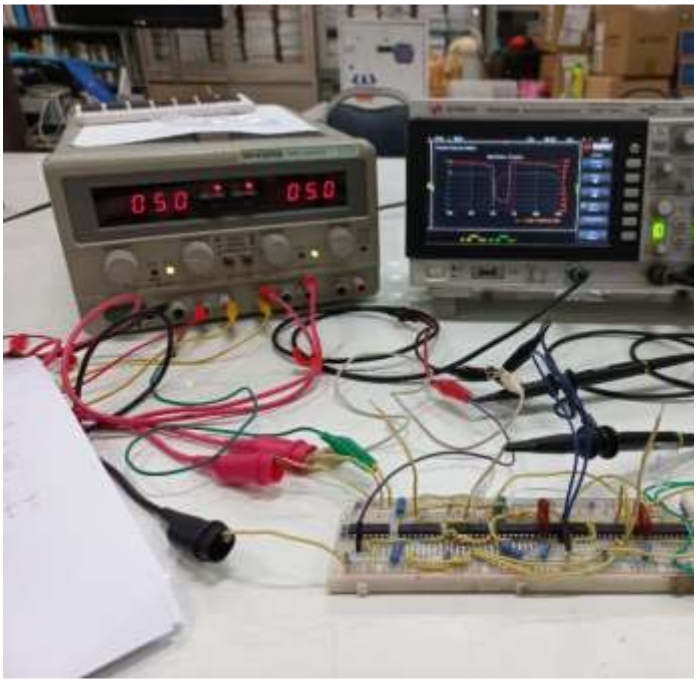

BP response of VM filter

BS response of VM filter

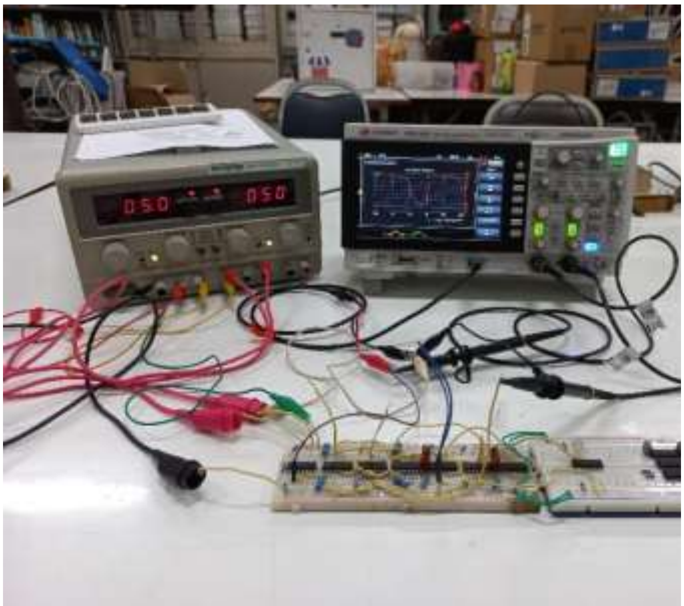

AP response of VM filter

Figure S1. Experimental setup of the universal filter.
